# Key Components of Human Myofibre Denervation and Neuromuscular Junction Stability are Modulated by Age and Exercise

**DOI:** 10.3390/cells9040893

**Published:** 2020-04-06

**Authors:** Casper Soendenbroe, Cecilie J. L. Bechshøft, Mette F. Heisterberg, Simon M. Jensen, Emma Bomme, Peter Schjerling, Anders Karlsen, Michael Kjaer, Jesper L. Andersen, Abigail L. Mackey

**Affiliations:** 1 Institute of Sports Medicine Copenhagen, Department of Orthopedic Surgery M, Bispebjerg Hospital, Building 8, Nielsine Nielsens vej 11, 2400 Copenhagen NV, Denmark; caspersoendenbroe@outlook.dk (C.S.); cjleidersdorff@gmail.com (C.J.L.B.); metteflindt@hotmail.com (M.F.H.); simonmarqvard@gmail.com (S.M.J.); emmabomme@gmail.com (E.B.); Peter@mRNA.dk (P.S.); ak@anderskarlsen.dk (A.K.); michaelkjaer@sund.ku.dk (M.K.); Jesper.Loevind.Andersen@regionh.dk (J.L.A.); 2 Xlab, Department of Biomedical Sciences, Faculty of Health and Medical Sciences, University of Copenhagen, Blegdamsvej 3, 2200 Copenhagen N, Denmark; 3 Center for Healthy Aging, Faculty of Health and Medical Sciences, University of Copenhagen, Blegdamsvej 3, 2200 Copenhagen N, Denmark

**Keywords:** sarcopenia, denervation, neuromuscular junction, heavy resistance exercise, acetylcholine receptor, cell culture, myogenesis, neonatal myosin, neural cell adhesion molecule

## Abstract

The decline in muscle mass and function with age is partly caused by a loss of muscle fibres through denervation. The purpose of this study was to investigate the potential of exercise to influence molecular targets involved in neuromuscular junction (NMJ) stability in healthy elderly individuals. Participants from two studies (one group of 12 young and 12 elderly females and another group of 25 elderly males) performed a unilateral bout of resistance exercise. Muscle biopsies were collected at 4.5 h and up to 7 days post exercise for tissue analysis and cell culture. Molecular targets related to denervation and NMJ stability were analysed by immunohistochemistry and real-time reverse transcription polymerase chain reaction. In addition to a greater presence of denervated fibres, the muscle samples and cultured myotubes from the elderly individuals displayed altered gene expression levels of acetylcholine receptor (AChR) subunits. A single bout of exercise induced general changes in AChR subunit gene expression within the biopsy sampling timeframe, suggesting a sustained plasticity of the NMJ in elderly individuals. These data support the role of exercise in maintaining NMJ stability, even in elderly inactive individuals. Furthermore, the cell culture findings suggest that the transcriptional capacity of satellite cells for AChR subunit genes is negatively affected by ageing.

## 1. Introduction

The rate of loss of muscle mass increases with advancing age [1], and ultimately leads to impaired physical function in elderly individuals [2,3,4]. This age-dependent decline in muscle mass is partly due to a loss of individual muscle fibres [5] as a result of muscle fibre denervation [6,7,8]. While physical exercise is recognized as a strong countermeasure against the loss of muscle mass and has consistently been shown to maintain physical function and health in the last ten years of life [9,10], it is currently unclear whether denervation can be ameliorated or reversed by exercise.

It has been shown in animals that exercise causes positive adaptations to the neuromuscular junction (NMJ) that to some extent can attenuate the age-related degeneration of the NMJ [11]. Changes in expression of acetylcholine receptors (AChRs) with acute exercise have been suggested to indicate NMJ remodelling in animals [12,13] and represent a potential target for studying this in humans [14]. AChR are present in abundance at the NMJ [15] and are almost non-existent in the extra-synaptic region of the muscle fibre [16]. Upon experimental denervation, however, the α1, β1, γ, and δ subunits increase extra synaptically [16,17,18,19], raising the possibility that these AChR subunits can be used as indicators of denervation associated with ageing. We recently observed a correlation between age and gene expression levels of the foetal γ AChR subunit in a large group (*n* = 70) of healthy elderly men ranging in age from 65 to 94 years, in conjunction with tissue markers of muscle fibre denervation, neural cell adhesion molecule (NCAM) and neonatal myosin (MHCn), at the protein level [20]. Direct comparisons with a younger cohort as well as the potential for exercise to influence AChR expression patterns are however lacking.

One of the challenges for ageing skeletal muscle is related to the decline in satellite cell function with age. Not only is satellite cell function important for tissue repair and maintenance, but it also has potential implications for maintenance of the NMJ, where myonuclei at this site must be capable of carrying out the specialization necessary to complete the formation of the NMJ. This includes producing a high concentration of AChRs at the membrane and a clustering of myonuclei, which become transcriptionally specialized and distinct from adjacent extra-synaptic myonuclei [21,22]. Whether this capacity declines with age is currently unknown. Satellite cells have been shown to play a vital role in maintaining the post-synaptic region in mice, both in terms of myonuclear clusters of AChRs and re-innervation of the regenerating NMJ [23,24]. In this context it is interesting that we have recently observed a poorer fusion capacity of satellite cells derived from old women compared to young women, accompanied by a distinctly different molecular profile throughout the myogenic program [25]. It remains unknown, however, to what extent this dysfunction in human satellite cells has implications for NMJ maintenance with increasing age.

Based on the above, the main purpose of this study was to investigate the influence of age and exercise on molecular markers of NMJ stability and muscle fibre denervation in healthy elderly individuals. An additional focus was to determine how ageing would alter the capacity of myonuclei in cell culture to produce key transcriptional elements for NMJ formation.

## 2. Materials and Methods

### 2.1. Experimental Design

This study is based on muscle biopsies collected from two studies, on 12 young and 12 elderly women [25], and on 25 elderly men [26], respectively. Both studies were approved by The Committees on Health Research Ethics for The Capital Region of Denmark (Ref: H-15017223, H-3-2012-081). All procedures conformed to the Declaration of Helsinki of 1975, revised in 2013, and the subjects gave written informed consent before participation. All participants were healthy, non-smokers, non-obese, and did not perform strenuous physical exercise on a regular basis. The men were part of a randomized controlled trial investigating the effect of the blood pressure-lowering medication losartan on the muscle response to exercise, where half of the participants received losartan and the other half placebo. Given the general lack of drug effect, the two groups were merged in the present study (separate group data are also provided for reference in online Supplementary Figure S1).

All participants performed a maximal strength test in a Leg Extension machine (M52, TechnoGym, Cesena, Italy) to determine the one-repetition maximum (1 RM), which was used to determine the load lifted during the subsequent bout of heavy resistance exercise. The Leg Extension exercise protocols consisted of both concentric and eccentric contractions. First, 4–5 sets of 12 concentric contractions at 70% of 1 RM were performed, followed by four sets of 4–6 eccentric contractions at 110% of 1 RM, as previously described [25,26]. The exercise was performed with one leg only, leaving the contralateral leg as a control. No other exercise was allowed during the study period.

### 2.2. Muscle Biopsies

For all participants, muscle biopsies were obtained from the vastus lateralis muscle, under local anaesthetic (1% lidocaine), using the percutaneous needle biopsy technique of Bergström [27], with five 6-mm needles and manual suction. Pieces of muscle tissue were aligned, embedded in Tissue-Tek, and then frozen in isopentane, pre-cooled in liquid nitrogen, and stored at −80 °C. The men had six muscle biopsies taken over 17 days, at the following time points: −10 and −3 days before exercise from the control, non-exercised leg, and from the exercised leg at +4.5 h and on days +1, +4, and +7 post exercise. The day −3 sample was excluded from the current study since its purpose was to investigate a potential effect of losartan in the rested state and is therefore superfluous in the current context. The young and elderly women had muscle biopsies collected from each leg five days after exercise, from which a part was embedded as described above and a part was used for cell culture, where myoblasts were plated in 12-well plates for three days of proliferation (12,000 cells per well), or three days of proliferation followed by four days of differentiation (20,000 cells per well), as previously described in detail [25].

### 2.3. RNA Extraction

100 cryo sections, 10 μm thick, from the embedded muscle tissue were homogenized in 1 mL of TriReagent (Molecular Research Center, Cincinnati, OH, USA) containing five stainless steel balls of 2.3 mm in diameter (BioSpec Products, Bartlesville, OK, USA), and one silicon-carbide sharp particle of 1 mm (BioSpec Products), by shaking in a FastPrep^®^-24 instrument (MP Biomedicals, Illkirch, France) at speed level four for 15 s. Cell culture cells were dissolved directly in the Trireagent. Bromo-chloropropane was added in order to separate the samples into an aqueous and an organic phase. Following isolation of the aqueous phase, RNA was precipitated using isopropanol. The RNA pellet was then washed in ethanol and subsequently dissolved in 20 μL RNAse-free water. Total RNA concentrations and purity were determined by spectroscopy at 260, 280, and 240 nm. Good RNA integrity was ensured by gel electrophoresis.

### 2.4. Real-Time RT-PCR

mRNA targets related to innervation were analysed for the current study. The specific primers are given in Table 1. Total RNA (500 ng for muscle and 150 ng for cell culture) was converted into cDNA in 20 μL using OmniScript reverse transcriptase (Qiagen, Redwood City, CA, USA) and 1 μM poly-dT (Invitrogen, Naerum, Denmark) according to the manufacturer’s protocol (Qiagen). The same pool of cDNA used previously for the cells in culture [25] and the male muscle tissue [26] was used here. For each target mRNA, 0.25 μL cDNA were amplified in a 25-μL SYBR Green polymerase chain reaction (PCR) containing 1 × Quantitect SYBR Green Master Mix (Qiagen) and 100 nM of each primer (Table 1). The amplification was monitored real time using the MX3005P Real-time PCR machine (Stratagene, San Diego, CA, USA). The Ct values were related to a standard curve made with known concentrations of cloned PCR products or DNA oligonucleotides (Ultramer^TM^ oligos, Integrated DNA Technologies, Inc., Leuven, Belgium) with a DNA sequence corresponding to the sequence of the expected PCR product. The specificity of the PCR products was confirmed by melting curve analysis after amplification. Ribosomal Protein Lateral Stalk Subunit P0 (RPLP0) mRNA was chosen as internal control. To validate this use, another unrelated “constitutive” mRNA, Glyceraldehyde-3-Phosphate Dehydrogenase (GAPDH), was measured and normalized with RPLP0. In the cell culture experiment GAPDH mRNA normalized to RPLP0 mRNA was constant, indicating that RPLP0 (and GAPDH) was indeed constant and suitable for normalization. However, in tissue the GAPDH/RPLP0 ratio was lower in the elderly female subjects and one and four days after exercise in the males, showing either a GAPDH decrease or a RPLP0 increase. However, the decrease in GAPDH was not reflected in the general pattern of the other mRNA when normalized to RPLP0, arguing against a general normalization error. We therefore chose to use retain RPLP0 for normalization. The GAPDH mRNA data from cell culture of the females and tissue of the males have been used as internal control in already published papers [25,26].

### 2.5. Immunohistochemistry

For the female participants, cross sections (10 µm) from the biopsies of the exercised and control legs were cut at −20 °C in a cryostat. Sections from both legs of one individual were placed on the same glass slide (Thermo Scientific, Waltham, MA, USA) and stored at −80 °C until staining. For staining, two primary antibodies were diluted in 1% bovine serum albumin (BSA) in Tris-buffered saline (TBS) and applied to the sections (see Table 2), and then incubated in the refrigerator overnight. Afterwards two secondary antibodies (see Table 2) diluted in 1% BSA in TBS were applied for 45 min. At this point, the sections were fixed in 5% formaldehyde (Histofix, Histolab, Gothenburg, Sweden) for 12 min and then mounted with Prolong-Gold-Antifade (Invitrogen, Molecular Probes, OR, USA, catalogue #P36931), containing 4′,6-Di-amidino-2-phenylindole (DAPI). Slides were washed with TBS twice between all steps. Slides were kept in darkness at room temperature for 48 h and then moved to a −20 °C freezer. Two sections were also stained with NCAM and collagen XXII (made by Manuel Koch) [28], as previously described [29], since it was suspected that the NCAM staining in these sections was due to the presence of myotendinous junction and not denervated muscle fibres.

### 2.6. Microscopy

All imaging was performed with a ×10/0.30NA objective and a 0.5× camera (Olympus DP71, Olympus Deutschland GmbH, Hamburg, Germany) mounted on a BX51 Olympus microscope, using the Olympus cellSens software (v.1.14). For all analyses, 1.7 × 1.3 mm greyscale images were captured.

Muscle fibre size and muscle fibre type composition analysis was only performed on the control leg. Non-overlapping images of high resolution (4080 × 3072 pixels) were captured to accommodate a semi-automated macro [30], run in ImageJ (v.1.51, U.S. National Institutes of Health, Bethesda, MD, USA). All analyses were conducted by the same person blinded to the age group. All included muscle fibres were manually checked, and fibres were excluded if the dystrophin staining was incomplete or if an area of the biopsy was longitudinally oriented. Fibres at the edge and around holes and folds in the biopsies were always excluded. After delineation of the muscle fibre cross-sectional area (CSA), fibre type was determined based upon the median light intensity. Fibres were classified as type I (positive for myosin type I staining) or type II (negative for myosin type I staining). Hybrid muscle fibres (low levels of type I myosin staining) were excluded from the analysis (a total of 131 fibres from all sections).

For the analysis of embryonic myosin heavy chain (MHCe)-, MHCn-, and NCAM-positive fibres, images at a resolution of 2040 × 1536 pixels were captured. For MHCe, only areas with positive staining were imaged, while for MHCn and NCAM the entire biopsy section was imaged (due to the relatively higher prevalence of positive fibres). Positively stained muscle fibres were determined as fibres with a complete dystrophin staining and a clear staining of one of the three markers. We extended the method used in our previous study [20] by also measuring the CSA of all transversely cut positive muscle fibres in the present study. All analyses were conducted by the same person, blinded to age group and leg of the sample. All values are expressed relative to the total number of fibres in the section. In a sub-analysis, four consecutive sections from two elderly subjects (both the exercised and the control leg) were additionally analysed for MHCn-positive fibres to determine whether small fibres could be found on consecutive sections. Overview images of the sections were initially used to identify areas of the biopsy that were present on all four consecutive sections. Peripherally positioned (at edges or holes) muscle fibres were not included. In total, 31 MHCn-positive muscle fibres were included across the two subjects and followed through the four consecutive sections (see online Supplementary Figure S2 for images).

### 2.7. Immunocytochemistry

For the cells cultured to differentiate, the fusion index was determined as reported earlier [25]. Briefly, coverslips were stained with the primary antibodies desmin and myogenin (see Table 2 for details) followed by the secondary antibodies goat anti-rabbit 568 (catalogue #A11036) and goat anti-mouse 488 (catalogue # A11029), and mounted with Prolong-Gold-Antifade containing DAPI (catalogue #P36931, Invitrogen), as described [25]. Fusion index was calculated as the percentage of desmin-positive nuclei within myotubes (containing three or more nuclei) divided by the total number of desmin-positive nuclei.

### 2.8. Statistics

All figures were prepared in GraphPad Prism (v.7.04, GraphPad Software, Inc., La Jolla, CA, USA) and all statistical analyses were conducted in SigmaPlot (v. 13.0, Systat Software Inc, San Jose, CA, USA), except subject characteristics and gene expression of the female subjects, which were analysed using Microsoft Excel 2016 (Microsoft Corporation, Redmond, Washington). *p*-Values below 0.05 were considered significant, and trends of *p* < 0.1 are also reported. mRNA data were normalized to RPLP0 and log-transformed before statistical analysis. For the female participants, unpaired *t*-tests (two-tailed) were performed between young and old for subject characteristics, fibre size, fibre type composition, and mRNA data. Paired *t*-tests (two-tailed) were conducted for the analysis of the exercise response (exercised leg vs. control leg). The Bonferroni correction was applied (multiplying the *p*-values ×3) to the *t*-test analyses on the mRNA data to correct for multiple testing. For correlation analyses, mRNA data were log-transformed and then subjected to Pearson’s correlation. The number of MHCe-, MHCn-, and NCAM-positive fibres, which was not normally distributed, was subjected to the Mann–Whitney Rank Sum Test and Wilcoxon Signed Rank Test to compare differences between young and old subjects, and control versus exercised leg, respectively. For the male participants, data were analysed by one-way repeated measures analysis of variance, using Dunnett’s method for multiple comparisons to compare each time point with baseline, where an overall main effect of time was found. The subject characteristics are presented as means with standard deviation and range, while muscle fibre size and composition are shown as individual values. MHCn- and NCAM-positive muscle fibres are presented as median and individual values.

## 3. Results

### 3.1. Subject Characteristics

Age, height, weight, BMI, and Leg Extension 1 RM for all subjects included in the analyses are provided in Table 3. The control muscle biopsy from one elderly woman was found to show irregularities (one fascicle filled with unusually large and small muscle fibres positive for NCAM, MHCn, and MHCe), and this subject was therefore excluded from all analyses.

### 3.2. Tissue Immunohistochemistry at Baseline—Young and Elderly Women

**Figure 1:**
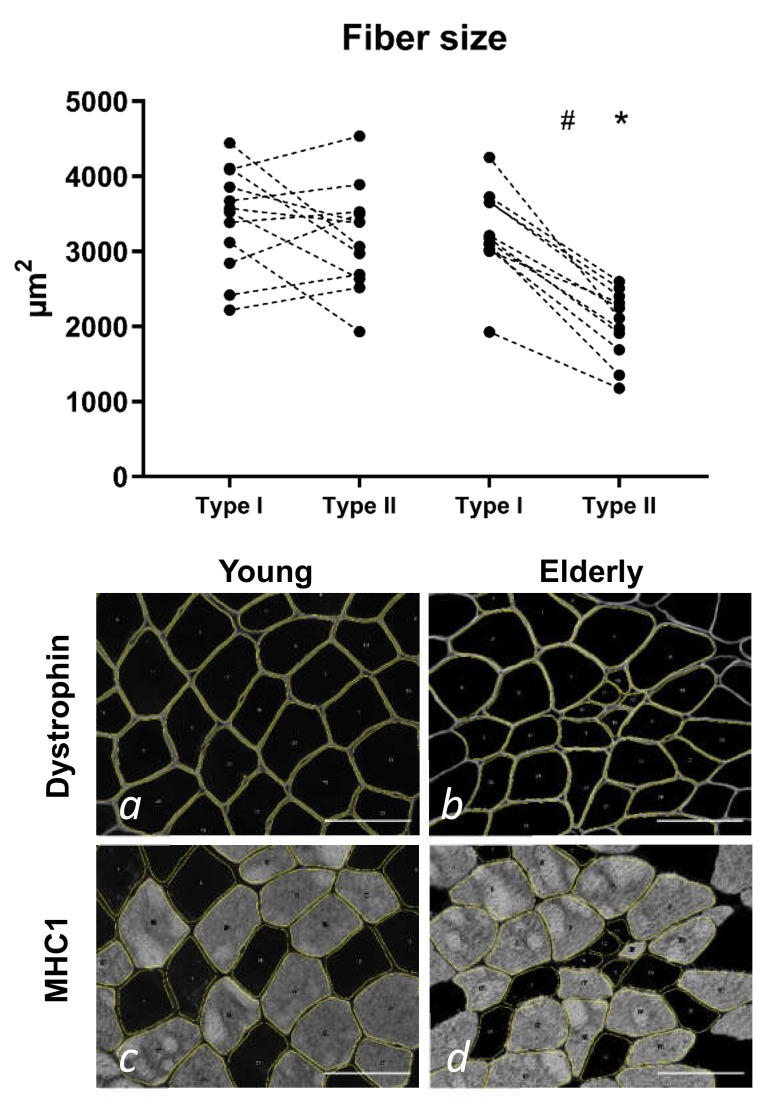
Muscle fibre size analysis in biopsy cross-sections from the control legs of 12 young and 11 elderly women. Individual values are displayed and with type I and II values for an individual connected by a dashed line. The type II fibres of the elderly individuals were significantly smaller than their own type I fibres and the type II fibres of the younger individuals. * *p* < 0.001 vs. young type II, # *p* < 0.001 between fibre types in elderly. Images (**a**–**d**) illustrate the analysis process. (b,d) show representative images of the same area, which has been delineated by the macro in ImageJ. This is an elderly subject with a mean fibre size of 3025 µm^2^ and 1688 µm^2^ for type I and II fibres, respectively. Similarly, a and c show representative images of the same area in a young subject with a mean fibre size of 3574 µm^2^ and 3378 µm^2^ for type I and II fibres, respectively. MHC1, myosin heavy chain 1. Scale bars = 100 µm.

On average, the numbers of fibres included in the fibre type and size analysis were 212 (129–352) for type I and 151 (68–247) for type II fibres in the young participants. The corresponding values for the elderly were 169 (85–267) type I and 143 (45–487) type II fibres. The type I fibre percentage was 59 ± 11% (35%–74%) for the young and 58 ± 15% (22%–75%) for the elderly, with no difference between them. As seen in Figure 1, the elderly had significantly smaller type II fibres compared to both their own type I fibres (−38%, *p* < 0.001) and the type II fibres in the young (−36%, *p* < 0.001).

**Figure 2:**
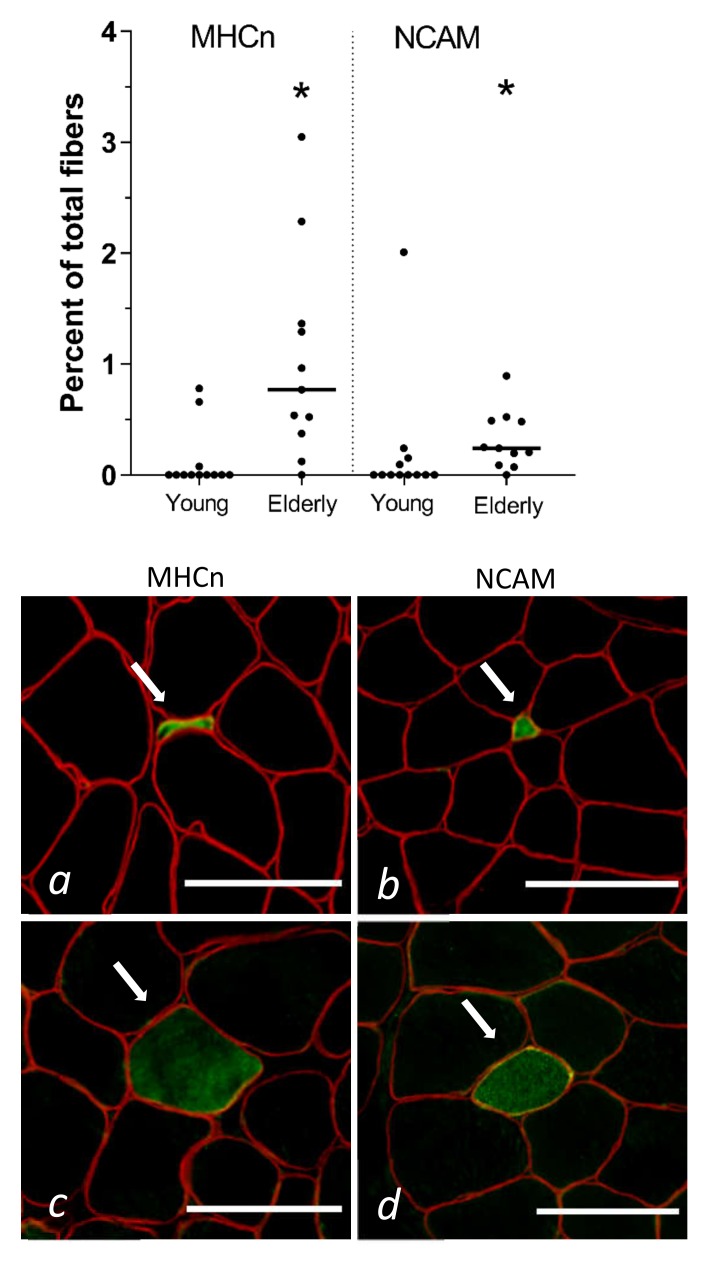
Muscle fibres positive for MHCn or NCAM in biopsy cross-sections from 12 young and 11 elderly women. Only the control leg is shown. Individual values are presented with the median (horizontal line). Panels show examples of small MHCn (**a**) and NCAM (**b**), and large MHCn (**c**) and NCAM (**d**) fibres (arrows). Positive fibres are green, dystrophin, red. * *p* < 0.05 vs. young. MHCn, neonatal myosin heavy chain; NCAM, neural cell adhesion molecule. Scale bars = 100 µm.

On average, the number of fibres included in the immunohistochemical analysis of denervated fibres was 1080 [401–2270]. MHCe-positive fibres were only found in the excluded subject and are therefore not presented. The elderly had significantly more MHCn- and NCAM-positive fibres compared to the young (Figure 2).

No significant differences between the previously exercised and the control leg were found in either the young or the elderly for MHCn or NCAM (online Supplementary Figure S3). We evaluated the fibre size of all transversely cut MHCn- and NCAM-positive fibres from the control leg. A clear majority of the MHCn- and NCAM-positive muscle fibres were smaller than 150 µm^2^ (online Supplementary Figure S3).

One biopsy from the exercised leg of a young subject showed 13 NCAM-positive fibres (1.3% of total fibre count) all located adjacent to a thick band of connective tissue, reminiscent of the myotendinous junction (MTJ). Collagen XXII staining confirmed that this was in fact MTJ, so these fibres were not included in the analysis of this biopsy (see online Supplementary Figure S4 for image). One young subject had 13 (1.45% of total fibre count) NCAM-positive fibres, all of which were located at the edge of the biopsy. This area was not stained by collagen XXII and remained NCAM-positive on additional sections and was therefore not excluded from the analysis. In all other samples MHCn- and NCAM-positive fibres were randomly scattered in between normal muscle fibres.

### 3.3. Tissue mRNA at Baseline and in Response to Exercise—Young and Elderly Women

**Figure 3:**
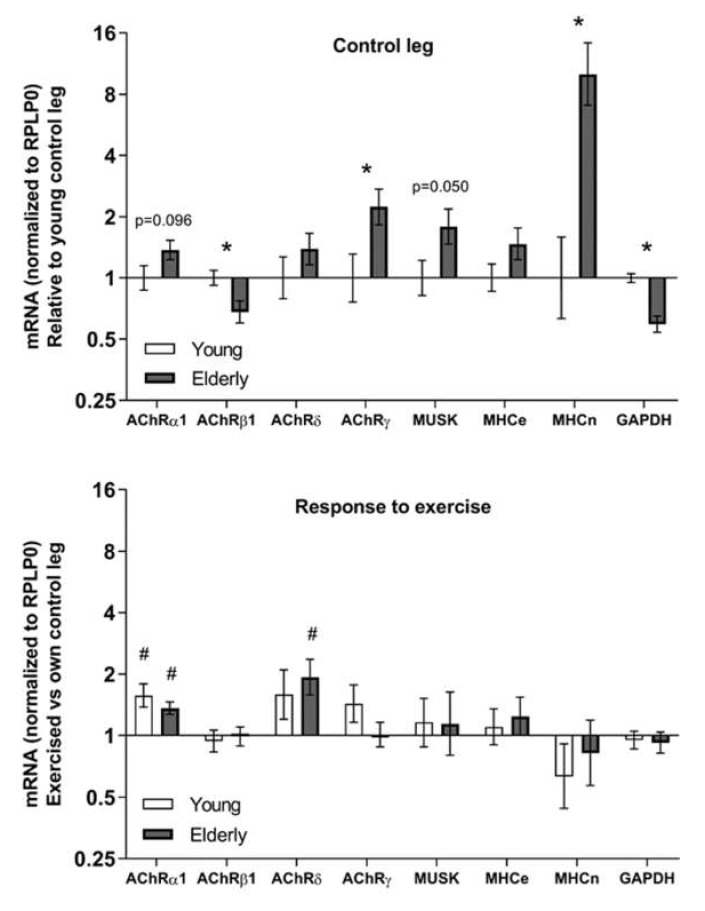
Gene expression in muscle biopsies of healthy young (*n* = 12) and elderly (*n* = 11) women, at rest (control) and five days after a single bout of one-legged exercise. mRNA data were normalized to RPLP0 and are shown as geometric means ± back-transformed SEM, relative to young control legs (control leg) and own control leg (response to exercise). * *p* < 0.05 elderly vs. young. # *p* < 0.05 vs. control leg. Tendencies are written. AChR: acetylcholine receptor; MuSK: muscle-specific-kinase; MHCe: embryonic myosin heavy chain; MHCn, neonatal myosin heavy chain; NCAM, neural cell adhesion molecule; GAPDH: Glyceraldehyde-3-Phosphate Dehydrogenase; RPLP0: Ribosomal Protein Lateral Stalk Subunit P0.

The muscle tissue of the elderly women had significantly lower levels of AChR β1 mRNA compared to the young women, whereas levels of both AChR γ and MHCn mRNA were higher in the elderly compared to the young (Figure 3). Tendencies for differences were seen for gene expression levels of AChR α1 and muscle-specific-kinase (MuSK).

Both the elderly and the young women had a significant upregulation of AChR α1 mRNA in the previously exercised leg compared to the control leg (Figure 3). The exercise response of AChR δ mRNA only reached statistical significance in the elderly. AChR ε mRNA were detected in less than half of the samples at levels very close to detection limit of one molecule and with no preference for any group (data not shown).

### 3.4. Cell Culture at Baseline and in Response to Exercise—Young and Elderly Women

The fusion index of the cell cultures from the rested and exercised legs of the elderly women was 36.3 ± 4.2% and 36.1 ± 5.0%, respectively, with the corresponding values for the young group being 52.2 ± 1.8% and 49.8 ± 2.2%, respectively (main effect of age, two-way repeated measures ANOVA).

**Figure 4:**
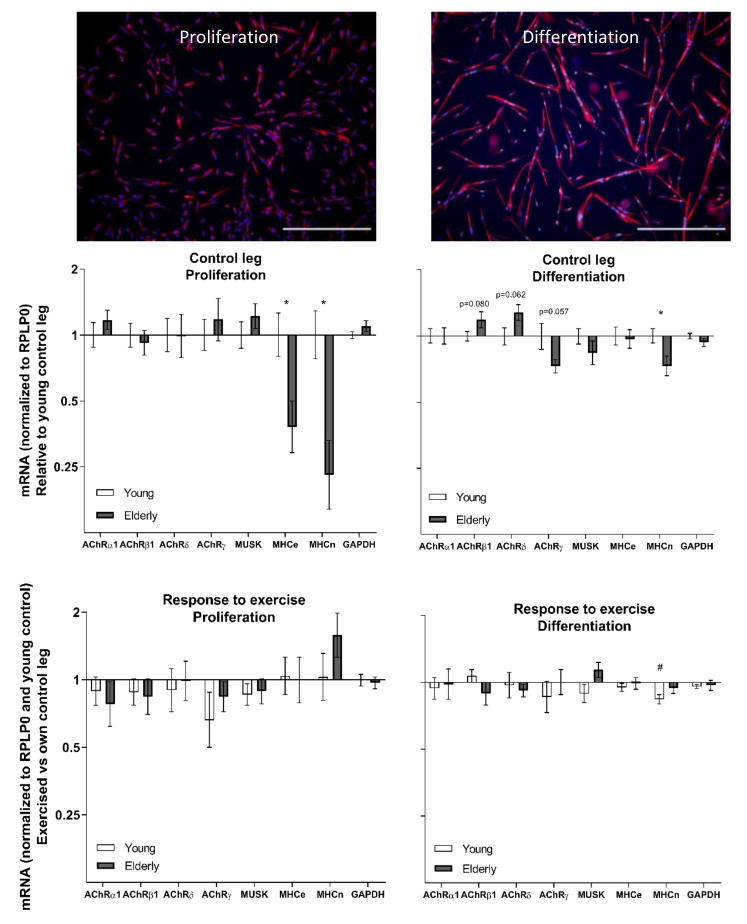
Images display cells in the proliferation condition (Desmin, red, and DAPI, blue) and in the differentiation condition (Desmin, red, Myogenin, green, and DAPI, blue), scale bars = 500 µm. Gene expression in cell cultures from the control and exercised legs of healthy young (*n* = 12) and elderly (*n* = 11) women. mRNA data were normalized to RPLP0 and are shown as geometric means ± back-transformed SEM, relative to young control leg (control leg) and own control leg (response to exercise). * *p* < 0.05 elderly vs. young. # *p* < 0.05 vs. control leg. Tendencies are written. AChR: acetylcholine receptor; MuSK: muscle-specific-kinase; MHCe: embryonic myosin heavy chain; MHCn, neonatal myosin heavy chain; NCAM, neural cell adhesion molecule; GAPDH: Glyceraldehyde-3-Phosphate Dehydrogenase; RPLP0: Ribosomal Protein Lateral Stalk Subunit P0.

All gene expression targets were more strongly expressed in differentiating compared to proliferating cells (see online Supplementary Figure S5). In the proliferating condition, the cells from the elderly had lower gene expression levels of MHCe and MHCn compared to young (Figure 4). Similarly, we also found a significantly lower level of MHCn gene expression in the differentiating cells in the control leg in the elderly compared to the young. AChR β1, δ, and γ all showed age-related tendencies.

Differentiating cells from the previously exercised leg from the young subjects demonstrated a lower gene expression of MHCn versus the control leg (Figure 4).

### 3.5. Tissue mRNA in Response to Exercise—Elderly Men

**Figure 5:**
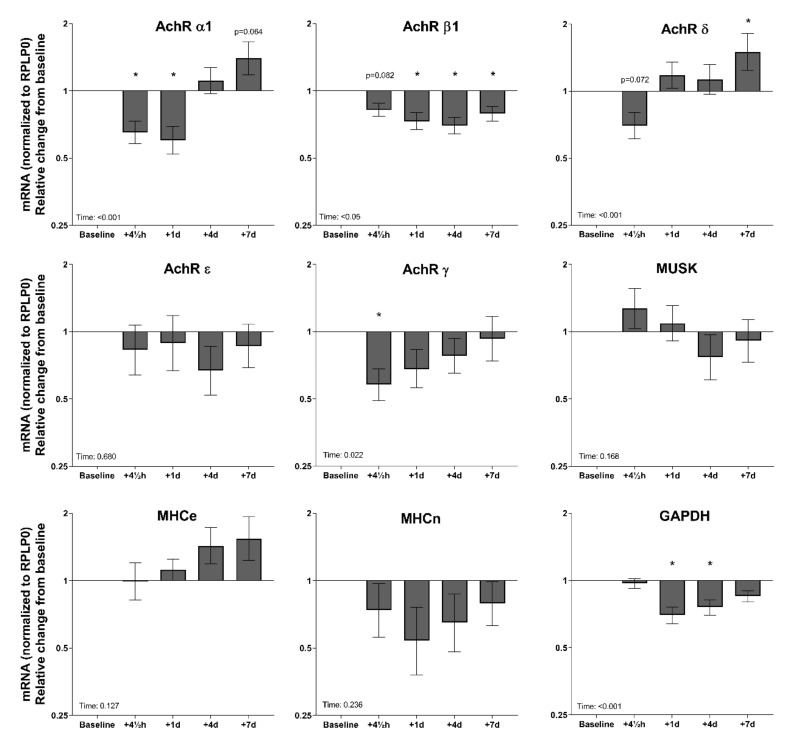
Gene expression in muscle biopsies of 25 healthy elderly men ten days before (baseline) and 4.5 h, one day, four days, and seven days after a single bout of exercise. mRNA data were normalized to RPLP0 and are shown as geometric means ± back-transformed SEM, relative to baseline. * *p* < 0.05 vs. baseline. Tendencies are written. AChR: acetylcholine receptor; MuSK: muscle-specific-kinase; MHCe: embryonic myosin heavy chain; MHCn, neonatal myosin heavy chain; NCAM, neural cell adhesion molecule; GAPDH: Glyceraldehyde-3-Phosphate Dehydrogenase; RPLP0: Ribosomal Protein Lateral Stalk Subunit P0.

In general, gene expression in four out of the five AChR measured demonstrated a response to exercise. AChR α1 mRNA was downregulated 4.5 h and one day after the exercise and returned to baseline in four days (Figure 5). AChR β1 mRNA was downregulated at 1, 4, and 7 days. AChR δ mRNA showed a tendency for a decline 4.5 h after exercise and was upregulated seven days after the exercise. AChR γ mRNA decreased 4.5 h after the exercise bout. No significant exercise-induced changes in gene expression of the AChR ε subunit, MuSK, MHCe, or MHCn were observed.

## 4. Discussions

The most notable findings of the present study was that the skeletal muscle of elderly individuals, with morphological signs of ageing as demonstrated by a reduced type II muscle fibre CSA and a heightened number of denervated muscle fibres, has a significantly elevated gene expression level of the denervation-responsive AChR γ subunit and MHCn as compared to young healthy individuals. Our data also suggest an age effect on the capacity of satellite cell-derived myotubes to transcribe AChR genes, which is fundamental for NMJ maintenance. Furthermore, we provide novel insight into the transient changes in gene expression of all five muscle AChR subunits following heavy resistance exercise in healthy elderly human skeletal muscle. Together these data support the role of exercise in stimulating the stability of the NMJ, but also indicate age-related changes, even in healthy elderly individuals.

### 4.1. Muscle Fibre Denervation in Elderly Humans

Healthy elderly women with clear signs of ageing (lower type II muscle fibre CSA and lower muscle strength), also show a significantly heightened number of denervated muscle fibres compared to young healthy women, as evidenced by a greater proportion of fibres positive for NCAM and MHCn. When a muscle fibre loses its neural input, the plasticity of the peripheral nervous systems allows for adjacent nerve sprouts to attempt to re-innervate denervated muscle fibres through nerve sprouting [31]. It is believed that the increased synthesis of NCAM in denervated muscle fibres facilitates this innervation process [32,33]. Denervated muscle fibres will also revert into an immature myosin heavy chain configuration, as we found more MHCn-positive fibres in old subjects compared to the young. Furthermore, we also observed a substantial 10-fold higher gene expression level of MHCn in the muscle tissue of the elderly compared to the young females, reflecting a persisting synthesis of this distinct myosin isoform. Importantly, it should be noted that there is not a complete overlap between MHCn- and NCAM-positive stained fibres, which suggests that the rate at which these proteins aggregate in the muscle fibres following denervation might differ. In terms of denervated muscle fibre morphology, we observed a persisting MHCn and NCAM protein presence in even the smallest of muscle fibres (<75 µm^2^). These miniature fibres are easily missed during regular biopsy assessments and could represent long-term denervated fibres that had atrophied over time [34] and undergone deterioration of muscle proteins [35], but maintained an increased and long-lasting cytoplasmic expression of MHCn [36] and NCAM [32]. The length of these miniature muscle fibres is a matter of uncertainty. We have previously been able to follow such fibres through 400 µm of consecutive biopsy sections in a selected subject [20]. In a sub analysis in the present study, we searched for MHCn-positive fibres through four consecutive sections and found that 13, 32, and 39% of the fibres had disappeared after 1, 2, and 3 sections, respectively. This implies a substantial number of miniature fibres ends, which could indicate that long-term denervated fibres are gradually degraded both transversally and longitudinally.

### 4.2. Ageing and Exercise Alter Acetylcholine Receptor Gene Expression

One of our most marked findings is that the gene expression of the AChR γ subunit is robustly elevated in the skeletal muscle of elderly compared to young females. This coincides with this subunit being a functionally distinct foetal subunit [37,38,39], which is increasingly expressed following both denervation [39,40] and neurotransmitter blocking [41]. Interestingly, in our group of male participants the muscle homogenate gene expression of the γ subunit was acutely downregulated after the exercise bout but had already returned to baseline after one day. We were also able to detect this subunit, as well as α1, β1, and δ subunits, in both proliferating and differentiating cell cultures that were devoid of neural presence, meaning that satellite cell derived myonuclei can upregulate AChR gene expression without the presence of a nerve. As satellite cells have been shown to be crucial for maintaining the specialized post-synaptic region of the muscle fibre [23], it is interesting that we observed trends for an age effect in three out of the four subunits. It is worth noting that this is the case even with the conservative Bonferroni correction, but given that the age difference was not always in the same direction, it is possible that this is a real effect of age (and not an effect of general cell culture conditions), potentially reflecting an age-related satellite cell dysfunction that could negatively impact the maintenance of the NMJ. However, it should be noted that the cell cultures derived from satellite cells of the young subjects showed a significantly higher fusion index compared to the old subjects [25], indicative of a higher level of myotube maturity. Furthermore, we also observed a positive correlation between cell fusion index and gene expression levels of AChRγ (R = 0.74), MuSK (R = 0.75), and MHCe (R = 0.66) in the old group (Supplementary Figure S6). This would suggest that AChRγ gene expression in aneural cell cultures is increased concordant with myotube maturity and raises the possibility that the molecular differences we observed between the cell cultures from young and elderly muscle are determined by the extent of fusion. However we cannot rule out the opposite, i.e., that the lower gene expression levels contribute to the lower fusion index values.

Generally, our data show age and exercise effects on AChR γ subunit gene expression, in line with its suggested use in evaluating muscle fibre denervation in healthy individuals. In our previous study, we found a negative correlation between age and the AChR γ subunit in a large group of elderly men [20], which initially might seem to contradict our finding in the present study. However, it is important to acknowledge that a denervated muscle fibre is not in a “stable state”, meaning that without the neural input the proteins will be degraded and the structure is gradually lost [34,35]. Ultimately, the muscle fibre completely disappears or is only present as a fraction of its former size and as such its contribution to the whole muscle gene expression profile will also decline.

Since our study includes one data set from males and the other from females, it is worth considering similarities in the pattern of the exercise response between the elderly male and female subjects, given that the day five timepoint of the females can be compared with the four- and seven-day timepoints of the male. In this way it seems that the α, δ, and γ subunits follow a similar pattern between the genders, with the first two subunits being upregulated in both male (α only a tendency) and female subjects after seven and five days, respectively, and the γ subunit being unaffected in both male and female subjects at these timepoints. The β subunit is consistently downregulated in the male subjects whereas this subunit is not affected in the elderly women five days after exercise. Whether this represents a true gender difference and what the functional significance might be however is unknown.

This study is to our knowledge the first to outline the gene expression time course for all AChR subunits following acute heavy resistance exercise and the first to analyse the expression of four out of five AChR subunits in both young and elderly individuals at rest and following acute exercise. The NMJ of humans is challenging to study molecularly since it is difficult to obtain actual human NMJs. Hence, we rely on extra-synaptic expression of various genes that are related to the NMJ. With this approach we observed that most subunits were found to be responsive to exercise, which would suggest that despite having reached an advanced age, there is a sustained tissue plasticity in terms of synthesizing new AChRs following an exercise stimulus. The subunit-specific responses also appear to be time-dependent, as some subunits were acutely reduced after exercise followed by a recovery phase, whereas others were downregulated for longer periods. The root of these widely diverging AChR subunit time courses is puzzling and, given evidence from animal studies that long-term exercise increases the size of the NMJ [11], it would be of interest to investigate the potential of lifelong exercise on NMJ adaptations in humans.

## 5. Conclusions

Taken together, these data support the concept that the loss of neural signal reverts certain muscle fibre proteins to an embryonic configuration (NCAM/MHCn/AChR γ) and that these markers are useful in evaluating the effectiveness of interventions to counteract the denervation-induced loss of muscle fibres in humans. Gene expression levels of the AChR γ subunit in particular repeatedly demonstrated sensitivity to age and exercise. The trends for age-related differences in the gene expression of AChR subunits in myotubes in cell culture were related to myogenic fusion index and potentially suggest a loss of satellite cell function in relation to the capacity to transcribe key molecules for NMJ stability. Finally, it can be speculated that the temporal manner of the AChR subunit gene expression response following exercise represents a beneficial stimulus for muscle mass preservation through strengthening of the NMJ.

## Figures and Tables

**Table 1:** Primers used for PCR. RPLP0: Ribosomal Protein Lateral Stalk Subunit P0; GAPDH: Glyceraldehyde-3-Phosphate Dehydrogenase; AChR: acetylcholine receptor; MuSK: muscle-specific-kinase; MHCn: neonatal myosin; MHCe: embryonic myosin heavy chain.

mRNA	Genbank	Sense	Antisense
RPLP0	NM_053275.3	GGAAACTCTGCATTCTCGCTTCCT	CCAGGACTCGTTTGTACCCGTTG
AchRα1	NM_000079.3	GCAGAGACCATGAAGTCAGACCAGGAG	CCGATGATGCAAACAAGCATGAA
AchRβ1	NM_000747.2	TTCATCCGGAAGCCGCCAAG	CCGCAGATCAGGGGCAGACA
AchRδ	NM_000751.2	CAGCTGTGGATGGGGCAAAC	GCCACTCGGTTCCAGCTGTCTT
AchRε	NM_000080.4	TGGCAGAACTGTTCGCTTATTTTCC	TTGATGGTCTTGCCGTCGTTGT
AchRγ	NM_005199.4	GCCTGCAACCTCATTGCCTGT	ACTCGGCCCACCAGGAACCAC
MuSK	NM_005592.3	TCATGGCAGAATTTGACAACCCTAAC	GGCTTCCCGACAGCACACAC
MHCe	NM_002470.3	CGGATATCGCAGAATCTCAAGTCAA	CTCCAGAAGGGCTGGCTCACTC
MHCn	NM_002472.2	CGGAAACATGAGCGACGAGTAAAA	CAGCCTGAGAACATTCTTGCGATCTT
GAPDH	NM_002046.6	GAGGGGCCATCCACAGTCTTCT	GACATGCCCAAGACCCAGAAGGA

**Table 2:** Antibodies used for immunohistochemistry and immunocytochemistry. MHCn: neonatal myosin; MHCe: embryonic myosin heavy chain; NCAM: neural cell adhesion molecule.

Host	Antibody	Primary Antibody Company	Cat. no.	Concentration
Mouse	Dystrophin, IgG2b	Sigma-Aldrich	D8168	1:500
Mouse	Myosin 1, IgG1	Hybridoma Bank	A4.951	1:200
Mouse	MHCe, IgG1	Hybridoma Bank	F1.652	1:100
Mouse	MHCn, IgG1	Novocastra	NCL-MHCn	1:100
Mouse	NCAM, IgG1	Becton Dickinson	347740	1:50
Rabbit	Desmin, IgG	Abcam	AB32362	1:1000
Mouse	Myogenin, IgG1	Hybridoma Bank	F5D-s	1:50
**Host**	**Antibody**	**Secondary Antibody Company**	**Cat. no.**	**Concentration**
Goat	488, green, IgG1	Invitrogen	A-21121	1:500
Goat	568, red, IgG2b	Invitrogen	A-21144	1:200
Goat	568, red, IgG	Invitrogen	A-11036	1:500
Goat	488, green, IgG	Invitrogen	A-11029	1:500

**Table 3:** Subject characteristics. Average and standard deviations with ranges (superscript). Abbreviations: BMI, body mass index; 1 RM, one-repetition maximum; yr: years, kg: kilogram.

	Young Women				Old Women				Old Men			
	*n* = 12				*n =* 11				*n = 25*			
Age (yr)	23	±	3	20–28	74	±	3	71–78	70	±	7	64–90
Height (cm)	168	±	7	157–177	166	±	3	162–169	180	±	5	172–189
Weight (kg)	64	±	8	53–75	69	±	10	57–84	82	±	10	67–98
BMI (kg/m^2^)	23	±	2	19–26	25	±	4	20–30	26	±	3	21–31
Knee extension 1RM (kg)	39	±	8	30–50	23	±	5	12–28	56	±	14	23–82

## Supplementary Materials

The following are available online at https://www.mdpi.com/2073-4409/9/4/893/s1, Figure S1: Gene expression in muscle biopsies of elderly men receiving losartan (*n* = 13) or placebo (*n* = 12). mRNA data were normalized to RPLP0, log-transformed, and are shown as geometric means ± back-transformed SEM, relative to baseline (–10d). Data were analysed with a two-way repeated measures ANOVA (treatment/time). * *p* < 0.05 vs. baseline. Tendencies are written; Figure S2: Panel showing four consecutive biopsy sections of an area with small MHCn-positive muscle fibres (a–d). The dotted squares (b,d) highlight the areas of the inserts (b_1 + 2_ and d_1 + 2_), in which a distinct dystrophin membrane is visible around the small fibre. Note that one of the positive muscle fibres is no longer visible in (c) and (d). MHCn-positive fibres are red, dystrophin, green, and nuclei, blue. MHCn, neonatal myosin heavy chain. Scale bars = 20 µm; Figure S3: Muscle fibre size of MHCn- and NCAM-positive fibres in biopsy cross-sections from the control leg in 12 young and 11 elderly women, pooled and plotted on a logarithmic scale (a). The majority of the positive fibres were <100 µm^2^.Muscle fibres positive for MHCn and NCAM for young and elderly women in control and exercised legs (b). No differences between control and exercised leg was observed for any variable. MHCn, neonatal myosin heavy chain; NCAM, neural cell adhesion molecule; Figure S4: Cross section of a muscle biopsy from one subject stained with NCAM (green) and collagen XXII (red). Nuclei are blue. NCAM-positive fibres are found in close proximity of a tendon-like structure and collagen XXII positivity confirms this is a myotendinous junction. These fibres were excluded from the analysis. Scalebar is 500 µm; Figure S5: Differentiating cells relative to proliferating cells in control leg of young women. mRNA data were normalized to RPLP0 and are shown as geometric means ± SEM. * *p* < 0.05 vs. proliferation; Figure S6: Myogenic fusion index correlates with cell culture mRNA levels of AChRγ, MuSK, and MHCe in rested leg of elderly (*n* = 10) but not young (*n* = 11) subjects. All mRNA data were log transformed and analysed with Pearson’s correlation, with R and P values displayed.

## Abbreviations

MHCe	Embryonic myosin heavy chain
MHCn	Neonatal myosin heavy chain
NCAM	Neural cell adhesion molecule
MHC1	Myosin heavy chain 1
DYST	Dystrophin
AChR	Acetylcholine receptor
DAPI	4′,6-Di-amidino-2-phenylindole
TBS	Tris-buffered saline
BSA	Bovine serum albumin
mTOR	Mammalian target of rapamycin
