# Supplementary material for: Key Components of Human Myofibre Denervation and Neuromuscular Junction Stability are Modulated by Age and Exercise
Source: Cells. 2020 Apr 6;9(4):893. doi: 10.3390/cells9040893 (PMC7226801; doi:10.3390/cells9040893)

Supplemental Figure 1

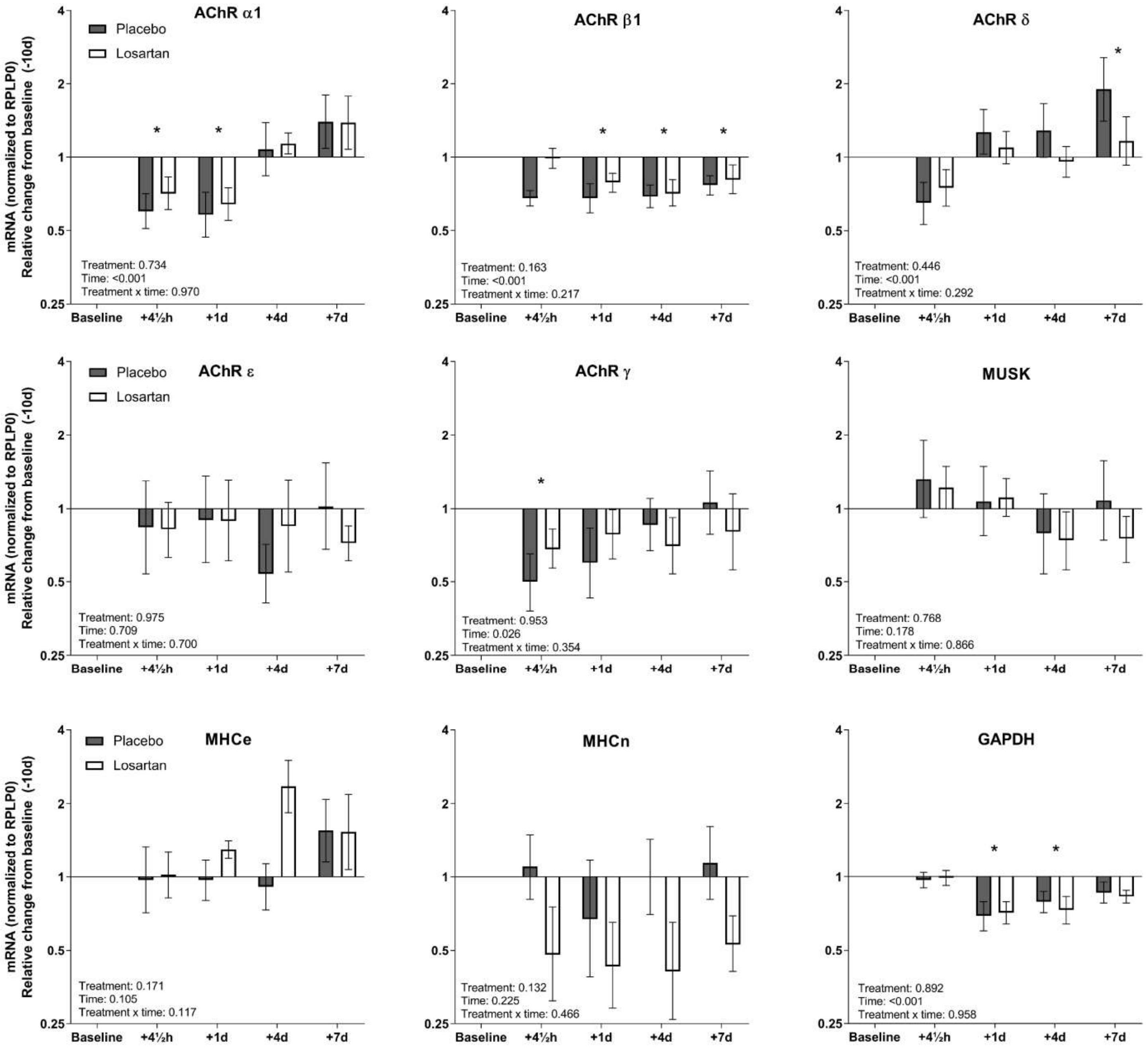

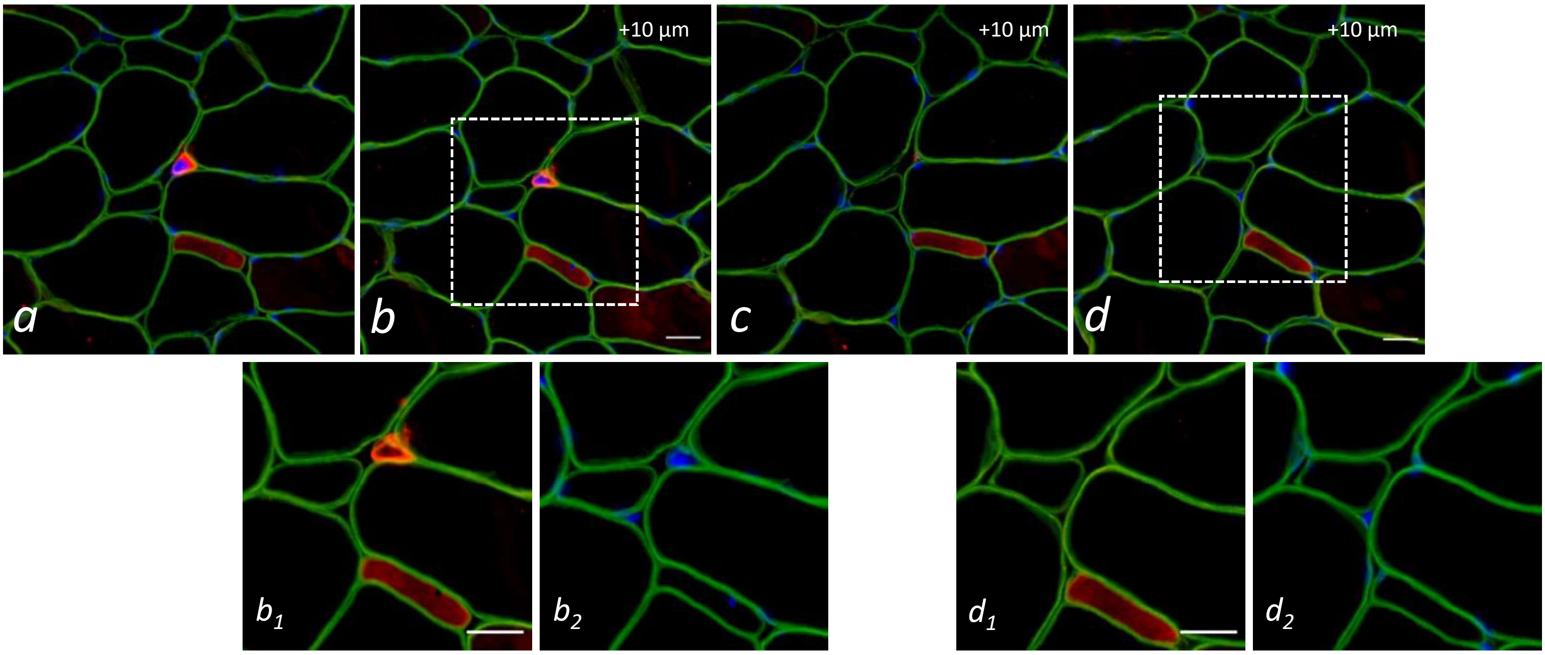

Supplemental Figure 2

Supplemental Figure 3

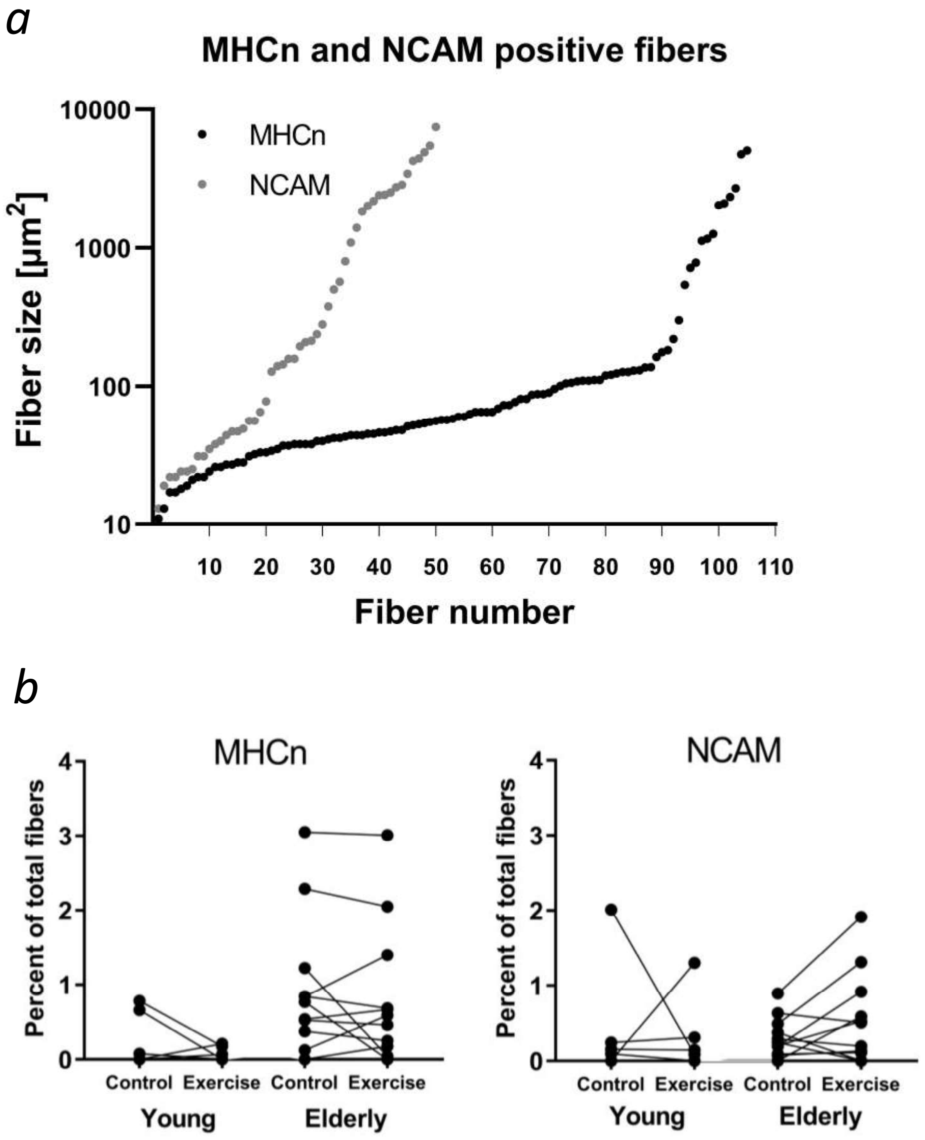

Supplemental Figure 4

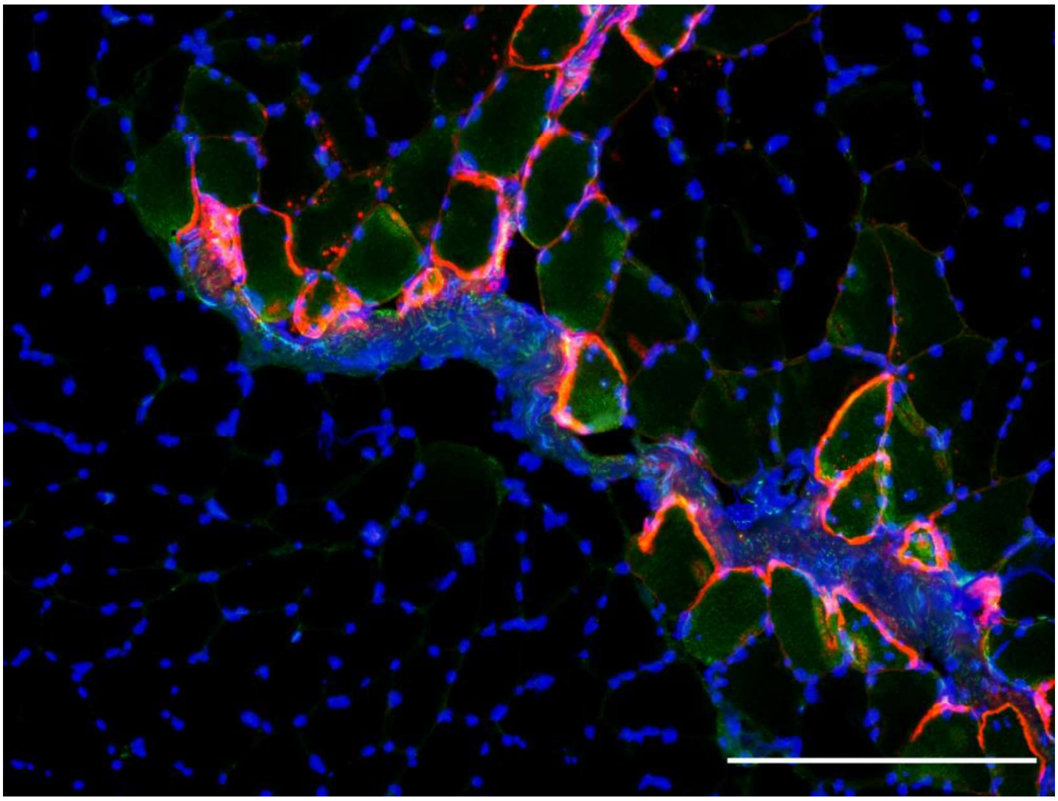

Supplemental Figure 5

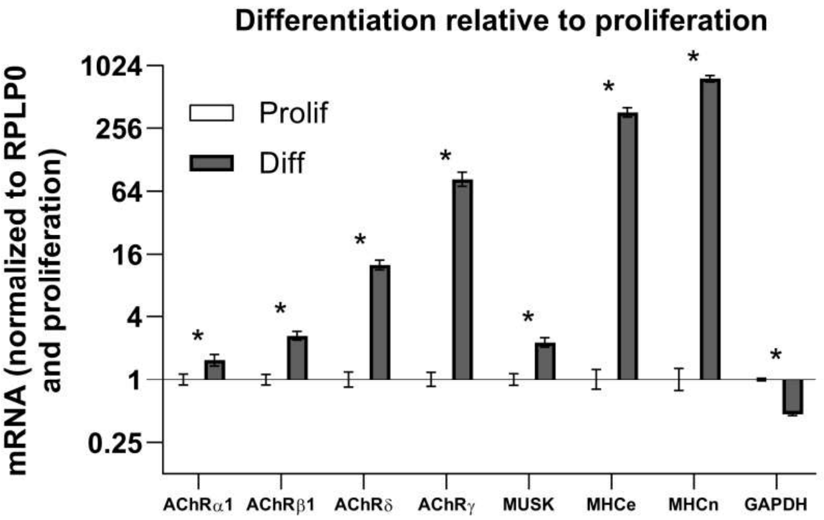

Supplemental Figure 6

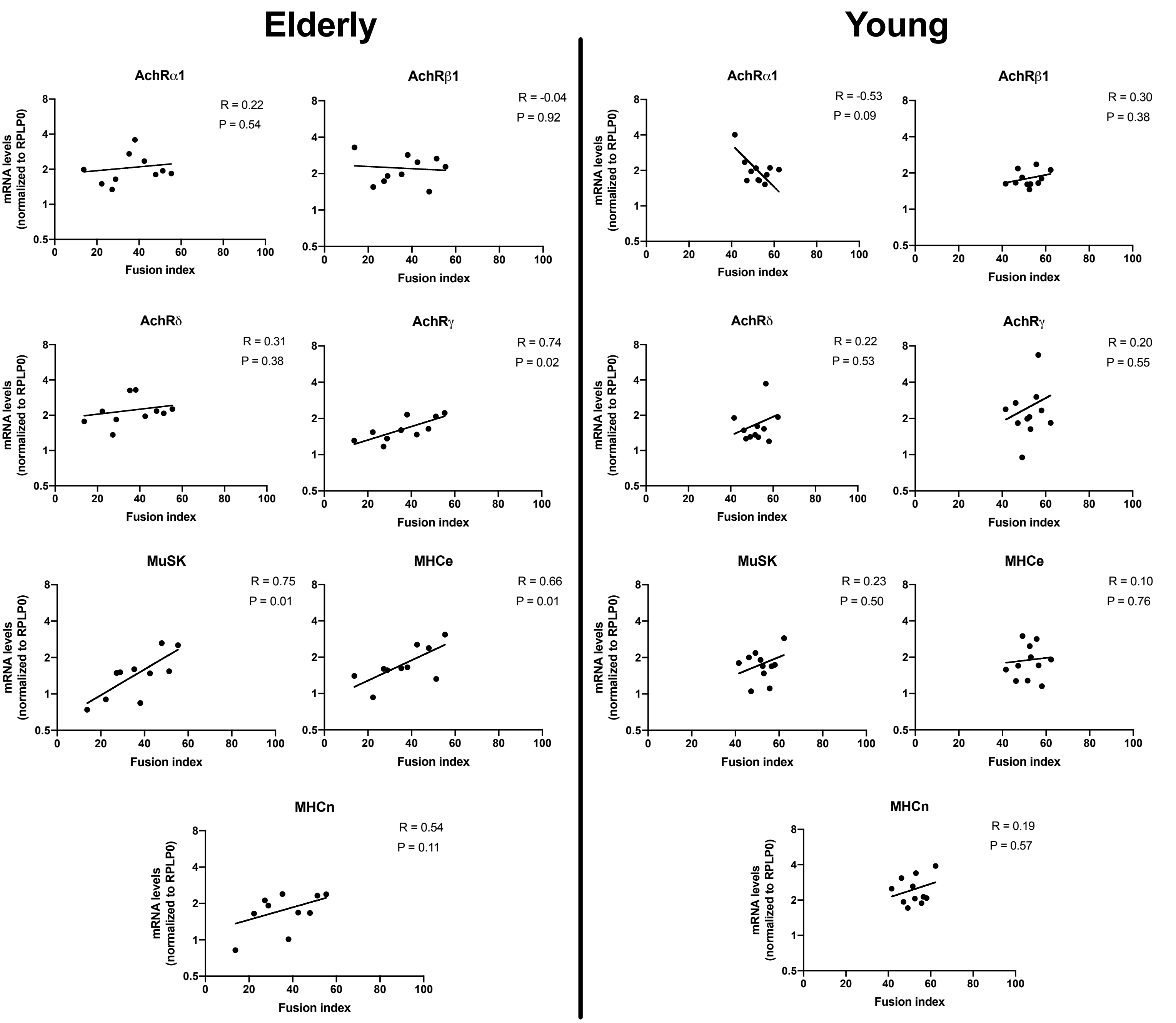

Supplement: Supplementary file 1 [file cells-09-00893-s001.pdf]
